# EZHIP: a new piece of the puzzle towards understanding pediatric posterior fossa ependymoma

**DOI:** 10.1007/s00401-021-02382-4

**Published:** 2021-11-11

**Authors:** Anne Jenseit, Aylin Camgöz, Stefan M. Pfister, Marcel Kool

**Affiliations:** 1 Hopp Children’s Cancer Center (KITZ), Im Neuenheimer Feld 280, 69120 Heidelberg, Germany; 2 Division of Pediatric Neurooncology, German Cancer Research Center (DKFZ) and German Cancer Consortium (DKTK), Heidelberg, Germany; 3 Faculty of Biosciences, Heidelberg University, Heidelberg, Germany; 4 National Center for Tumor Diseases (NCT), Dresden, Germany; 5 Department of Hematology and Oncology, University Hospital Heidelberg, Heidelberg, Germany; 6 Princess Máxima Center for Pediatric Oncology, Utrecht, The Netherlands

**Keywords:** PFA ependymoma, EZHIP, PRC2, H3K27M, DMG, H3K27me3

## Abstract

Ependymomas (EPN) are tumors of the central nervous system (CNS) that can arise in the supratentorial brain (ST-EPN), hindbrain or posterior fossa (PF-EPN) or anywhere in the spinal cord (SP-EPN), both in children and adults. Molecular profiling studies have identified distinct groups and subtypes in each of these anatomical compartments. In this review, we give an overview on recent findings and new insights what is driving PFA ependymomas, which is the most common group. PFA ependymomas are characterized by a young median age at diagnosis, an overall balanced genome and a bad clinical outcome (56% 10-year overall survival). Sequencing studies revealed no fusion genes or other highly recurrently mutated genes, suggesting that the disease is epigenetically driven. Indeed, recent findings have shown that the characteristic global loss of the repressive histone 3 lysine 27 trimethylation (H3K27me3) mark in PFA ependymoma is caused by aberrant expression of the enhancer of zeste homolog inhibitory protein (EZHIP) or in rare cases by H3K27M mutations, which both inhibit EZH2 thereby preventing the polycomb repressive complex 2 (PRC2) from spreading H3K27me3. We present the current status of the ongoing work on EZHIP and its essential role in the epigenetic disturbance of PFA biology. Comparisons to the oncohistone H3K27M and its role in diffuse midline glioma (DMG) are drawn, highlighting similarities but also differences between the tumor entities and underlying mechanisms. A strong focus is to point out missing information and to present directions of further research that may result in new and improved therapies for PFA ependymoma patients.

## Ependymomas

Ependymomas (EPN) are central nervous system (CNS) tumors that can arise in the supratentorial brain (ST-EPN; covering the cerebral hemispheres), hindbrain or posterior fossa (PF-EPN; including the cerebellum and brainstem), or anywhere in the spinal cord (SP-EPN) [43]. They can occur across all ages but are about 2.5 times more common in children, where they account for 5–6% of all malignant brain tumors, than in adults [12, 85]. The 10-year overall survival (OS) is 50–73% in pediatric patients, increases by patient age and reaches up to 80% in adults [22, 89]. Moreover, in children below the age of 3, the 5-year OS rate drops further down to 42–55% [22, 55, 64]. In children, 90% of EPN occur intracranial, with two-thirds of these cases located in the hindbrain [39, 57].

Breakthroughs in the biology of ependymomas and what is driving them came from molecular studies, including DNA methylation and transcriptional profiling, indicating that ependymomas in the different anatomical compartments of the CNS are biologically different [34, 57]. The nine molecular groups, three in each compartment, identified by DNA methylation profiling, are also transcriptionally highly distinct [57]. Most importantly, these analyses clearly demonstrated that an objective risk stratification by molecular profiling is superior to a risk stratification by histological grading [57]. A poor outcome with high relapse rate was only observed for two groups, the infratentorial PFA group and the supratentorial ST-EPN-RELA group, most recently re-annotated as ST-EPN-ZFTA as *ZFTA* (aka *C11orf95*) and not *RELA* is the most common fusion partner in this group [56, 90]. More recently, two groups independently described another small but distinct group of spinal ependymomas characterized by *MYCN* amplifications and poor outcome [23, 73], which brings the total to 10 molecular groups of ependymomas that were all included in the new 5th edition of the WHO classification of CNS tumors [43]. Sequencing studies have shown that supratentorial ependymomas are largely driven by fusion genes, including *ZFTA-RELA* in ST-EPN-ZFTA and *YAP1-MAMLD1* in ST-EPN-YAP1 tumors [57, 58, 90]. Recent studies analyzing even larger series of supratentorial ependymomas demonstrated that there is still quite some heterogeneity within these molecular groups and other oncogenic fusions, almost all involving *ZFTA*, have been identified within the ST-EPN-ZFTA tumors [90]. In addition, for the ST-EPN-YAP1 group, other fusion partners of *YAP1* have been identified [74]. However, except for a few rare genomic rearrangements (*MYCN* amplification, and *CDKN2A* deletions), no fusions or other highly recurrent mutations have been identified in other ependymoma groups that could explain their distinct biology, but several recent studies have provided more insight in other oncogenic mechanisms that drive the posterior fossa A ependymomas, which are the focus of this review.

## Posterior fossa ependymomas

DNA methylation and transcriptional profiling of posterior fossa ependymomas have identified three distinct molecular groups, annotated as PF-EPN-A (or PFA), PF-EPN-B (or PFB), and PF-SE, where the last group is enriched with cases histologically classified as subependymomas [20, 44, 57, 82]. All three groups come with different clinical characteristics. While PFA ependymomas are a disease of young children (median age 3 years with 58% < 4 years, 41% 4–18 years, 1% > 18 years), PFB and PF-SE ependymomas are more common in adolescents, young adults and adults (PFB: median age 30 years, with 19% 4–18 years and 81% > 18 years; PF-SE: median age 59 years and 100% > 18 years) [57]. In addition, clinical outcome is very different between the three groups, with the worst outcome seen for PFA ependymomas (10 years OS 56%). In contrast, patients with PFB or PF-SE tumors do much better with a 10 year OS of 88% and 100%, respectively. Gain of chromosome 1q, a well-established marker for poor outcome in ependymoma, is enriched among PFA ependymomas [4, 40, 57, 66]. Interestingly, 1q gain also identifies a group of patients within the PFA group for whom the outcome is worse than for PFA tumors without 1q gain [56, 57], whereas in PFB tumors, 1q gain does not seem to be associated with a worse outcome [20]. In addition, loss of chromosome 6q has been identified as another predictive factor in PFA ependymomas, identifying a group of patients at very high risk [7, 41, 54].

**Fig. 1:**
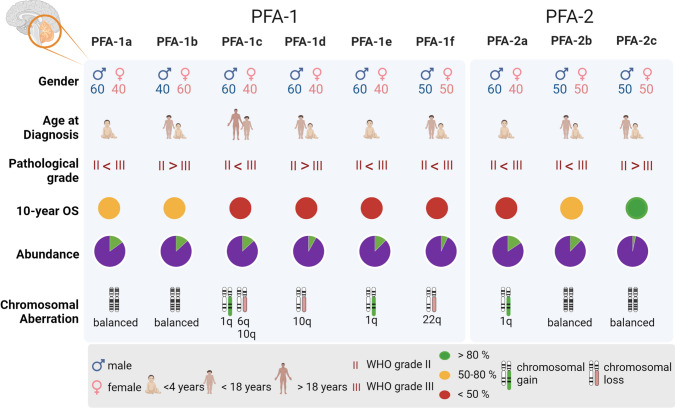
Clinical characteristics of PFA ependymoma subtypes. PFA ependymomas are divided into six PFA-1 and three PFA-2 subtypes. Characteristics shown are the gender distribution of patients, their average age at diagnosis, the occurrence of WHO grades II and III, the 10-year overall survival (OS), the abundance of the subtype within PFA ependymomas and the most occurring chromosomal aberrations

DNA methylation analysis of larger series of posterior fossa ependymomas revealed further heterogeneity within the PFA and PFB groups, identifying two major subgroups (PFA-1 and PFA-2) and nine distinct subtypes (PFA-1a-e and PFA-2a-c) within PFA (Fig. 1) [56], and five distinct PFB subtypes (PFB1-5) [20], all with distinct demographics, copy number alterations and transcriptional profiles. While no significant differences in outcome were observed for the PFB subtypes, and also not between the two major PFA subgroups, the outcome between the nine PFA subtypes differed significantly. PFA subtypes associated with a very poor outcome included PFA-1c, which is highly enriched for cases with 1q gain, PFA-1d, and PFA-1e (10 years OS 42, 40 and 44%, respectively). In contrast, PFA-2c tumors, characterized by high levels of OTX2 expression, which is not seen in any of the other PFA ependymomas, are associated with a very good outcome (10 years OS 95%) [56].

RNA sequencing has not identified any recurrent fusion transcripts in posterior fossa ependymomas. In addition, initial whole-genome and whole-exome sequencing approaches revealed an extremely low overall mutation rate in both PFA and PFB tumors and found no relevant recurring somatic single-nucleotide variants (SNV) [58], suggesting that these tumors are most likely driven by alternative and possibly epigenetic mechanisms [58]. This initial hypothesis was further supported by later in-depth sequencing efforts on larger series of PFA ependymomas in which rare but recurrent mutations were identified in epigenetic proteins like enhancer of zeste inhibitory protein (EZHIP) and Histone H3 (H3) [56]. Moreover, whole-genome DNA methylation analyses showed that PFA and PFB have very distinct DNA methylomes [44]. For instance, promoter cytosine-phosphate-guanine (CpG) islands were found to be hypermethylated in PFA compared to PFB ependymomas. As this seemed to come with a downregulation of epigenetically regulated genetic programs in PFA, epigenetic control aberrations were suspected to play a role especially in PFA tumorigenesis. This was supported in the same study showing that the global distribution of repressive histone mark histone 3 lysine 27 trimethylation (H3K27me3) across the genome differs between PFA and PFB ependymomas, and that differential H3K27me3 marks could be used to distinguish them [44]. The initial conclusion, however, that the CpG hypermethylation and the differential H3K27me3 levels were caused by an overly active Polycomb Repressive Complex 2 (PRC2), responsible for setting the H3K27me3 marks, now seems to be overturned by more recent studies. As DNA methylation and histone methylation are closely linked in epigenetic regulations, Bayliss et al. investigated H3K27 and CpG island methylation in ependymomas and demonstrated the inverse relationship between CpG island and H3K27 methylation. In other words, PFA with high CpG island methylation reveals global reduction in H3K27me3 levels compared to PFB [9]. Altogether, these findings strengthened the impression that PFA ependymomas are driven by epigenetic changes in DNA and histone methylation.

## EZHIP in PFA ependymomas

The global loss of H3K27me3 in PFA ependymomas strongly suggested epigenetic mechanisms as tumorigenic drivers of this disease. However, H3K27M mutations that cause low H3K27me3 levels in diffuse midline gliomas (DMG), are rare in PFA ependymomas and have been identified in only 4.2% of cases [50, 56, 67]. In our series [56], H3K27M mutations were limited to 13/310 PFA cases (4.2%), which all belong to the PFA-1 subgroup, but were highly enriched in the PFA-1f subtype (69%; 9/13). Instead, we and others identified EZHIP (previously known as CXorf67) as the main responsible protein for the diminished H3K27me3 levels in PFA ependymomas [31, 32, 56]. EZHIP is expressed in almost every PFA ependymoma, but not in cases that harbor the H3K27M mutation or in any of the other EPN groups.

**Fig. 2:**
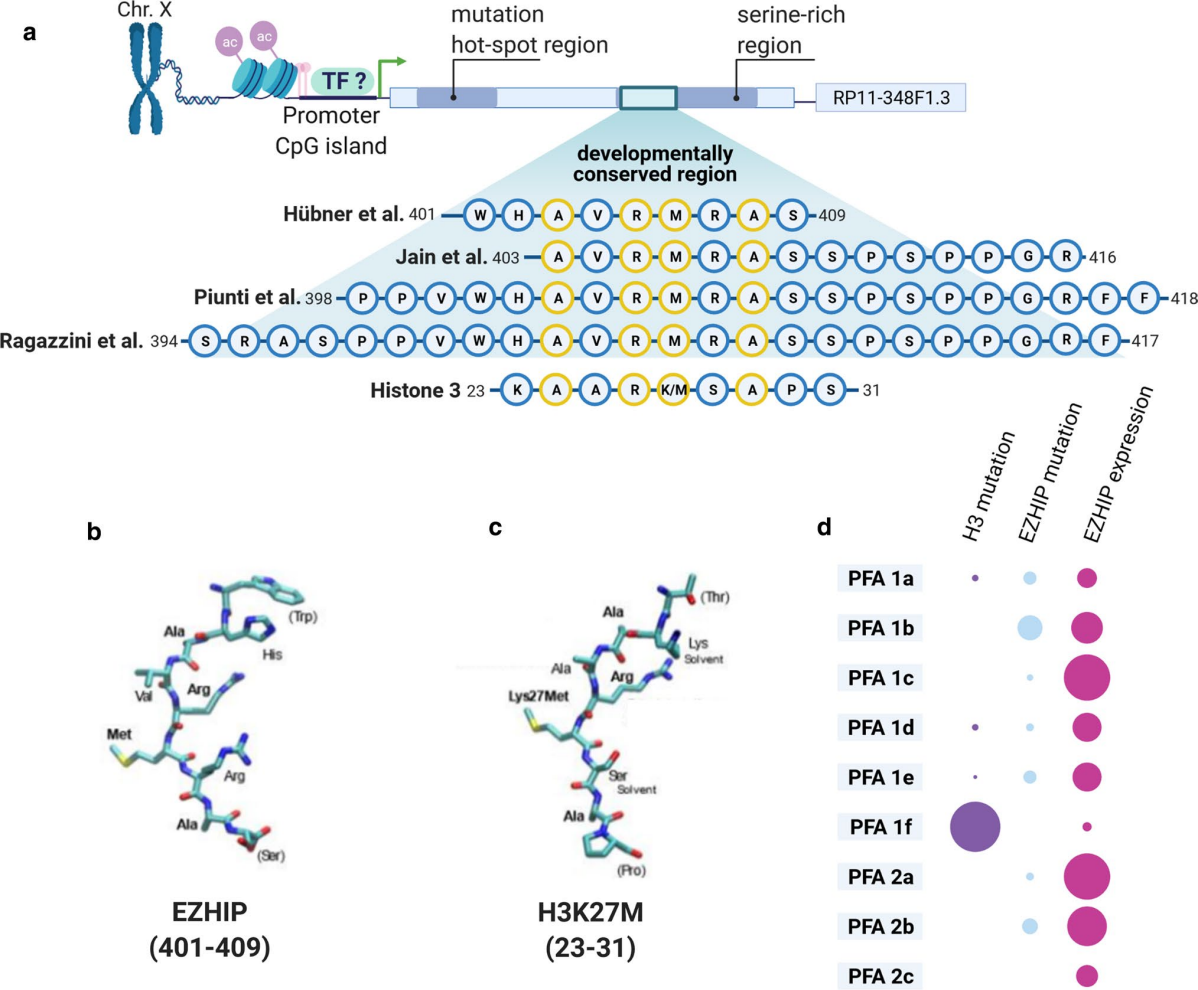
The relationship of EZHIP and H3K27M. **a** Schematic gene structure of EZHIP on chromosome X. Zoom in to the developmentally conserved region and the consensus region as it was used in different publications. Alignment with the tail region of histone 3 shows the high similarity to the H3K27M motif, with perfectly matched amino acids in yellow. **b**, **c** Amino acid structure of the EZHIP consensus region (**b**) and the H3K27M oncohistone region (**c**) when folded into the EZH2 binding pocket, adapted from Hübner et al. [30]. **d** Comparison of histone 3 mutations, EZHIP mutations and EZHIP expression levels between the PFA subtypes showing the mutual exclusivity of histone 3 mutations with EZHIP expression

In other CNS tumors, *EZHIP* is not expressed except for a small group of CNS germ cell tumors [56]. Recent reports also described DMGs with elevated *EZHIP* expression in cases that lack H3 mutations, which is in line with the mutual exclusivity between *EZHIP* expression and H3 mutations seen in PFA (Fig. 2) [13, 63]. Due to this high specificity, EZHIP expression is discussed as a simple but reliable prognostic IHC biomarker for PFA ependymomas and EZHIP expressing DMGs [2, 49]. Outside the CNS, EZHIP expression is found in endometrial stromal sarcoma (ESS) [16] and squamous non-small cell lung cancer (NSCLC) [17].

The fact that *EZHIP* is expressed only in this small subset of tumors is considered the reason for its late recognition, even though we already described it in 2015 as part of a PFA signature [57]. Only few experiments have been performed in the limited number of available PFA cell lines, as they are generally slow growing and difficult to culture [56]. A recent study on the metabolism of PFA ependymomas suggested that lower oxygen levels improves in vitro growth [46]. Moreover, in this study, it was shown that hypoxia drives the expression of *EZHIP* and the *PRC2 modulators Elongin BC and Polycomb repressive complex 2-associated protein* (*EPOP*) in primary PFA cultures. Together with an increase in catabolic processes such as glycolysis, glutaminolysis and reductive carboxylation of glutamine, inhibited PRC2 activity contributes to a modulated epigenetic state of the cells, resulting in a growth benefit in cell culture [46]. Independent of the oxygen status, EZHIP expression, like many others, is regulated via its promoter CpG island methylation. As such, hypomethylated *EZHIP* promoters are found in PFA tumors but not in other posterior fossa ependymomas [62]. PFAs with H3K27M mutations showed an almost twofold higher methylation of the *EZHIP* promoter than tumors with wild-type H3*,* in accordance with the mutually exclusivity of H3K27M mutations and EZHIP expression in PFA and DMG [13, 56, 63]. Non-PFA cancer cell lines with EZHIP expression include the Daoy cell line, for a longtime presumed to be a medulloblastoma cell line, and the osteosarcoma U2OS cell line [32, 62]. If expressed, EZHIP protein localizes mainly to the nucleus, but can be detected in cytoplasmic fractions as well [31].

Until today, it is not clear whether EZHIP is naturally expressed during PFA tumor initiation in the cell of origin or becomes activated in the process of tumorigenesis. The latter seems to be the case in squamous NSCLC [17] and might seem likely for PFA, too, as the only healthy tissues to express EZHIP are oocytes, testis and ovaries, but not the adult brain [76]. We can only speculate about the expression of EZHIP during brain development, as the cartography of expression in the developing fetal brain is far from complete.

A retrospective analysis of sequencing data of 30 PF ependymomas identified somatic mutations in *EZHIP* in five PFA tumors. Targeted sequencing of another 234 PFA tumors revealed single-nucleotide variants (SNVs) in 22 tumors (9.4%), with mutations detected in seven out of the nine PFA subtypes; not in PFA-1-f and PFA-2-c. Only three SNVs were present in more than one tumor and the majority of all mutations identified (68%) was located in a hotspot region between codons 71 and 122 [56, 58]. *EZHIP* mutations do not correlate with clinical or pathological parameters, nor do they seem to influence *EZHIP* expression levels. Across other tumor entities, *EZHIP* mutations are rare. The highest frequency (5.8%; *n* = 599) is found in ESS, a tumor entity frequently comprising fusion genes involving PRC2 components [16, 56]. Interestingly, one case-based study of ESS reported two tumors where EZHIP acted as 3`-fusion partner for Malignant Brain Tumor Domain Containing 1 gene (MBTD1). Both fusions included the functional serine-rich region of EZHIP [16], which further highlights the universal function of EZHIP, and of the serine-rich region in particular.

### The structure of EZHIP

The human *EZHIP* gene is nested into the introns 1–2 of the RP11-348F1.3 non-coding gene at Xp11.22 [16]. In a single exon, *EZHIP* comprises an open reading frame of 1512 bases, coding for a 51 kDa protein of 503 amino acids. It does not contain any common domains, but instead is predicted to be intrinsically disordered. The SNV hotspot region at the N-terminus, however, might be of order and may contain a potential protein–protein interaction domain, implicating functional consequences of the mutations [56]. However, until today it is unclear how the mutations in EZHIP affect the function of the protein or what their role is in ependymoma tumorigenesis.

The *EZHIP* gene is present only in placental mammals and conservation between species is low. The exception is a highly conserved short consensus motif within the serine-rich region towards the C-terminus. Depending on the study and how stringent the consensus motif was defined, it stretches somewhere between amino acids 398 to 418 [31, 32, 62, 65]. Interestingly, this region always includes a shorter motif of highly conserved amino acids that match (even though not perfectly) the amino acids 23–31 of H3. This H3 sequence includes the often post-translationally modified or mutated K27 matching the methionine M406 of EZHIP [31, 32, 62]. As the H3K27M mutations increase the sequence homology to EZHIP, the consensus motif has also been called K27M-like peptide (KLP) [32].

H3K27M (but not H3 wt) interferes with the function of EZH2 [42]. Intriguingly, the conserved region of EZHIP binds the catalytic site of EZH2 in a highly comparable way. The consensus motif of EZHIP even is remarkably close to a previously calculated “optimal” EZH2 target sequence, which would perfectly fit the catalytic preferences of EZH2. In EZHIP, this similarity is reinforced by the amino acid at the -1 position to the crucial lysine (M406 in EZHIP) that is an arginine as preferred by EZH2. This R405 of EZHIP directly interacts with the EZH2 residues D652 and Q648 via salt bridges. Further interactions with EZH2 residues are most likely performed by non-identical, but similar enough, amino acids in the consensus motif [5, 31, 32].

### The function of EZHIP

After the first report of its mutation and overexpression in PFA ependymomas, increased attention sparked the start of the ongoing functional characterization of EZHIP not only in PFA [31, 32], but also in ESS [62] and germ cells [65].

The natural role of EZHIP during development, as suggested by its expression pattern, might be a rather small one, as *Ezhip* knockout (KO) mice showed no developmental defects or abnormalities and adults were not distinguishable from wild-type (wt) mice [65]. Males (-/Y) were fertile with only little effect of the *Ezhip* KO on spermatozoa mobility. Female fertility, on the other hand, was impaired age-dependently. Homozygous KO females had smaller ovaries than heterozygous (+/-) mutants and wt mice. The stronger effect of *Ezhip* KO on females might be rooted in the almost 4 times higher expression of *Ezhip* in ovaries compares to testis, but may also be influenced by the localization of *Ezhip* on chromosome X, as it will be silenced in spermatocytes during meiotic sex chromosome inactivation [65]. However, this does not seem to translate to human cancer. So far, no sex bias on clinical prevalence, outcome or EZHIP expression was detected in PFA or DMG patients [36, 56].

In cultured cells, depletion of EZHIP reduces growth and increases elimination but shows only little effect on the growth of engrafted Daoy cells [26, 56]. Effects in culture were smaller than of the elimination of EZH2 [62], which goes in line with the results of a CRISPR-screen in primary PFA cells that did not identify *EZHIP*, but *EZH2* and other PRC2 components, as essential genes for growth in PFA [46]. It thus seems that EZHIP keeps the activity of the PRC2 complex at the crucial level, which is needed for PFA tumorigenesis while at the same time inhibiting EZH2 enough to change gene expression. This proposed “Goldilocks-Model” of balance between inhibition and activity again highlights the importance of PRC2 in PFA and might be indicative for future therapeutic approaches targeting the epigenetic nature of these tumors.

Indeed, EZHIP is not the primal single player keeping this delicate balance of PRC2 as global gene expression changes conferred by EZHIP were smaller than initially expected. Single-oocyte RNA sequencing revealed deregulation of only 100 genes upon KO. *EZHIP* KO in human U2OS cells led to changes in genes described by the gene ontology (GO) terms nucleosomes, DNA packaging and extracellular space [65]. In contrast, the overexpression of EZHIP in HEK293 led to the deregulation of genes associated with neurogenesis, enzyme linked receptors, NS development or regulation of cell differentiation. The serine-rich region, including the consensus region, is sufficient and essential to convey these expressional changes [31]. The main mode of action of EZHIP to convey these changes still seems to be via the PRC2 complex, as deregulated genes are often PRC2 target genes and overlap with genes sensitive to H3K27M expression or PRC2 component deletion [31, 32]. However, EZHIP does not interfere with the expression of PRC2 components themselves or their association with each other [62, 65].

In addition, recent data suggested a potential role of EZHIP independent of the PRC2 complex. EZHIP was found to interact with participants of the homologous recombination (HR)-mediated DNA repair pathway, preventing the smooth function of the PALB2-BRCA2 axis. Upon DNA-damage, EZHIP localizes to the damage sites and ultimately prevents the resolution of DNA double strand breaks. Having a motif (aa 420–432) similar to the part of BRCA2 that interacts with the WD40 domain of PALB2, EZHIP competes with BRCA2 and prevents it from being recruited to the damage sites, thereby interrupting the HR process [26]. From many other cancers, impaired HR capacity (most famously due to mutations in the *BRCA* genes) is a known sensitizing factor for the use of PARP inhibitors due to synthetic lethality [70]. In their study, Han et al. thus continued to also test the effect of PARP inhibition on EZHIP expressing cells and (non-PFA) PDX models. Even though their results seem promising, more experiments need to be done in PFA cells and tumor models, as their biology is crucially different from cancer cell lines like Daoy or U2OS. The limited penetration of the blood brain barrier (BBB) by PARP inhibitors is an additional concern that needs to be tackled by future research including PFA-specific pharmacokinetic and pharmacodynamic characterizations [27, 51, 72, 86, 87].

### EZHIP reduces H3K27me3 via EZH2

**Fig. 3:**
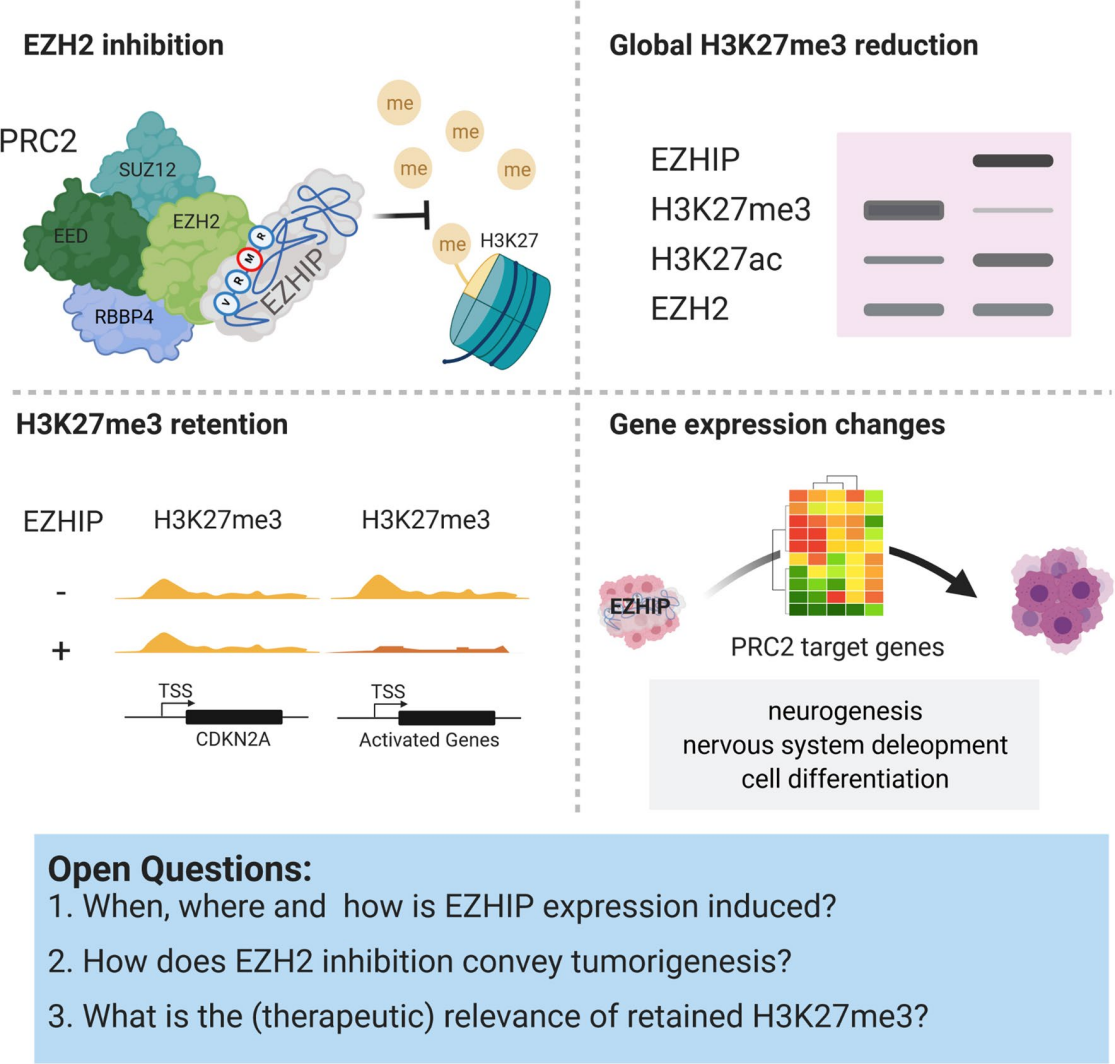
Downstream effects of EZHIP expression. EZHIP inhibits EZH2 in the PRC2 complex thereby reducing the repressive H3K27me3 mark. The loss of H3K27me3 activates gene expression, but specific genes (e.g., CDKN2A) retain the H3K27me3 upon EZHIP expression. Global gene expression changes conveyed by EZHIP expression resemble PRC2 target gene repression

As EZHIP itself probably does not possess an enzymatic function, the identification of functional interaction partners was the focus of early research. EZHIP was shown to interact with PRC2 components in different compositions in multiple publications (Fig. 3). Immunoprecipitation followed by mass-spectrometry (IP-MS) not only identified all PRC2 enzymatic core components (EZH2, SUZ12, EED, and RBBP4) as EZHIP interaction partners, but also PRC2 associated proteins (e.g., JARID2, MTF2) [32, 56, 65]. Antibody-based detection confirmed the direct interaction of EZHIP with EZH2 and SUZ12. EED might not directly interact with EZHIP, but it seems that its presence increases the association of EZHIP with EZH2 [31, 33, 62]. The interaction with EZH2 and SUZ12 is conveyed by the C-terminal region of EZHIP. In contrast, the interaction with RBBP4 is mediated by the N-terminal region of EZHIP and its interaction with JARID2 or AEBP2 depends on the SET domain of EZH2 [31, 62]. In addition, one study connected the interaction of EZHIP with PRC2 core members to the presence of the EZH2 co-factor S-Adenosyl methionine (SAM) [32].

EZHIP also inhibits EZH1, the paralog of EZH2, via interaction with the protein but with a lower affinity for EZH1 than for EZH2 [32]. In a competitive mode of inhibition, EZHIP prevents the methylation of H3K27 dose-dependently. While the allosteric activation of EZH2 via H3K27me3 binding to EED is not disturbed, it seems that EZHIP prevents the PRC2 complex from spreading the H3K27me3 mark after the initial recruitment to chromatin [32, 33, 35]. Two studies revealed a stronger inhibitory potential (lower IC50) for EZH2 of EZHIP than of H3K27M peptides [31, 32]. Interestingly, the minimum consensus sequence around M406 seems insufficient to inhibit EZH2 alone. The degree of extension of this sequence needed to enable full inhibition, just as the definition of the consensus sequence itself, is dissimilar between reports even though all confer the same basic message. Hübner et al. report a slight increase of four amino acids N-and C-terminally each (V400-P420) to be necessary and sufficient for an EZHIP peptide to reach the inhibitory potential of the commercial EZH2 inhibitor GSK126 [31]. On the other hand, Jain et al. find that their KLP (A403-R423) is a strong PRC2 inhibitor in cell-free assays, but fails to inhibit PRC2 in HEK293T cells, even if extended to stretch amino acids 317–423 or 395–423. The additions of serine-rich intrinsically disordered repeats (IDRs) either C- or N-terminally of the consensus sequence, predicted to mediate protein–protein interactions, seem to overcome this shortcoming successfully. Even though not potent alone, the central role of M406 as a H3K27 mimic becomes clear upon mutation. The inhibitory effect of EZHIP diminishes upon mutation of M406 into a basic (M406K or M406R) or not-acidic amino acid (M406E) [32]. Mutation into isoleucine (M406I), on the other hand, does not affect the inhibitory potential, and M406K even converts EZHIP into an EZH2 target. Additional mutations in the EZHIP hotspot region never interfere with the inhibitory function of EZHIP [32].

Globally, the inhibition of EZH2 by EZHIP translates into altered post-translational modifications (PTMs) on H3K27. PFA tumor tissue is characterized by low H3K27me3 and H3K27me2 levels. In contrast, H3K27me1 levels are higher in PFA than in ST-EPN, as is H3K27 acetylation (H3K27ac), an activating mark in competition with H3K27me3 [46]. The reduction of H3K27me3 (up to 80%) in PFA tissue is especially strong in intergenic regions and retained marks are characterized by smaller, sharper peaks in Chromatin-Immunoprecipitation followed by sequencing (ChIP-seq) experiments [32]. This is independent of the PFA identity, as cell lines of any background expressing EZHIP (endogenously or exogenously) show the same phenotype of low H3K27me2/3 levels and increased H3K27ac. H3K27me3 marks are gained as sharp peaks around transcriptional start sites (TSS), correlating with chromatin occupation by SUZ12 and PRC2 target gene repression [65]. *EZHIP* KO in U2OS or Daoy cell lines reverses this effect without affecting related marks set by PRC1, like H3K27me1 or H2Aub [56, 62, 65]. It can be assumed that a removal of EZHIP in PFA would show the same effects, but experimental proof is still pending.

Some loci, however, retain the repressive H3K27me3 mark in PFA, even upon EZHIP overexpression. At these loci, ChIP-Seq peaks are of smaller width as it can be observed for instance at the *CDKN2A* locus. The tumor suppressor gene is kept under repression in PFA, H3K27M positive gliomas, as well as in EZHIP expressing cell lines [32, 62]. The criteria for a locus to remain repressed by H3K27me3 upon EZH2 inhibition are still under investigation. Identifying a pattern would be of great power, as releasing tumor suppressors by therapy presents a potential treatment option against these tumors.

## Learning from the oncohistone H3K27M in DMG

Diffuse intrinsic pontine gliomas (DIPG), more recently now included in the category known as DMG, are deadly pediatric malignancies found in the brain stem. Like PFA ependymomas, they are characterized by a midline location, young patients, bad outcome and global loss of H3K27me3, in their case mainly caused by the oncohistone H3K27M. As mentioned earlier and similar as in PFA, H3K27M mutations in DMG are 100% mutually exclusive with EZHIP expression [13, 56]. Understanding the mechanisms of how H3K27M mutations and EZHIP drive tumorigenesis in PFA and DMGs may form a base to create better therapeutic options for the patients.

However, in contrast to PFA, DMGs harbor a variety of genetic aberrations in different combinations [45]. In addition to mutations in H3, DMGs are characterized by p53 loss-of-function (LOF) mutations in 40–50% of tumors. Receptor tyrosine kinase (RTK) pathways are commonly affected, with amplifications or activating mutations of *platelet-derived growth factor receptor alpha* (*PDGFRA*) in 30 or 5% of cases, respectively [37, 59, 60]. Other common findings are six different somatic mutations of *activin receptor type 1A* (*ACVR1*) detected in 21–32% of DMG patients [75, 84]. The genetic background of DMGs is thus clearly different from PFA.

Just like EZHIP in PFA, H3K27M is the defining marker of DMGs and significantly worsens patient overall survival. However, H3K27M is also not the sole driver of DMG tumorigenesis and acts tumorigenic only when supported by the aforementioned mutational background [38, 45, 71]. This fact is mirrored in how genetic mouse models for DMGs are created. For mice to develop DMG tumors, additional genetic events as well as the correct location and time point of gene delivery or induction are crucial, showing the necessity for a correct biological background [19, 25, 48, 61]. Histone methylation is especially variable during development and might be an essential confounding factor in the cell of origin [47]. So far, neither the precise cell of origin nor additional hits to EZHIP are known for PFA tumorigenesis. Thus, identification of the correct target cell for genetic manipulations will be a crucial step forwards in modeling PFA ependymomas [24, 79].

### H3K27M and EZHIP act background independently

Independent of the affected H3 variant (heterozygous mutations can occur at different variants of H3: *HIST1H3B*, *HIST1H3c*, and *H3F3A*), expression of H3K27M directly results in a global reduction of H3K27 methylation in the tumor tissue. H3K27me3 is lost especially in intergenic regions, but specific sites, such as the *CDKN2A* locus, retain their marks, and activating H3K27ac marks are unaffected or unchanged [10, 14, 28, 42, 78, 83]. This re-distribution of PTMs on H3 is highly reminiscent of the effect of EZHIP in PFA and indicates that the effects of H3K27M and EZHIP are independent of tumor cell context [28, 42].

With the shared mechanism of EZH2 inhibition, the effects of EZHIP and H3K27M on gene expression are much alike: both PFA and DMGs show an overall de-repression of PRC2 target genes [28, 31, 62, 65]. Their general moderate effect on gene expression is highest on H3K27me3-silenced or lowly expressed genes [28, 32]. Experiments with DMG further characterized many of them as genes with bivalent promoters (H3K4me and H3K27me3 positive), which are often involved in developmental processes [11, 69]. If this is caused by the nature of PRC2 targets and not a characteristic of the cell of origin of DMGs, one should expect similar results for PFA. Regardless of all the mechanistic similarities between DMG and PFA and independent of the expression of H3K27M or EZHIP, their overall expression profiles and DNA methylation characteristics are still very distinct [13], most likely due to a different cellular origin and the presence of additional tumorigenic events and mutations in DMG tumors.

### Common targets in DMG and PFA

Despite their inhibition, PFA and DMG tumors both heavily rely on PRC2 core components to sustain proliferation. Independent experiments showed that tumor cells of both entities are sensitive to inhibition of EZH2 and EED in vitro. In addition to the identification of PRC2 components as essential genes in PFA, they also react to a lack of the EZH2 co-factor SAM. *EZH2* KO prolongs the survival of DMG tumor bearing mice and proliferation can be impaired by a SUZ12 knockdown. The effects seem to rely on the presence of H3K27M in DMG, independent of the affected H3 variant [46, 48, 61]. Taken together, it seems that the residual PRC2 activity after inhibition is a necessity in PFA and DMG for tumor maintenance. This further highlights the importance of genes that retain their H3K27me3 marks and might be a possible starting point for therapy.

The two-faced role of EZH2 in PFA and DMG—repressed but also essential in its residual activity—fits the overall highly ambiguous role of EZH2 in cancer [18]. In some entities, like T cell Acute Lymphoblastic Leukemia (T-ALL) or lung adenocarcinoma, EZH2 fits the role of a tumor suppressor and is found deleted or inactivated by mutations [53, 80]. In contrast, EZH2 overexpression and activating mutations are reported for prostate, breast, gastric cancer and others [6, 21, 77]. So far, a clear understanding of what determines whether EZH2 acts oncogenic or tumor suppressive is missing. PFA and DMG research should benefit from increased knowledge in other entities and might act as a two-in-one model system at the same time. As different EZH2 inhibitors are already being tested for a variety of epigenetically driven tumors, their use might prove useful in PFA and DMG in the future [18].

**Fig. 4:**
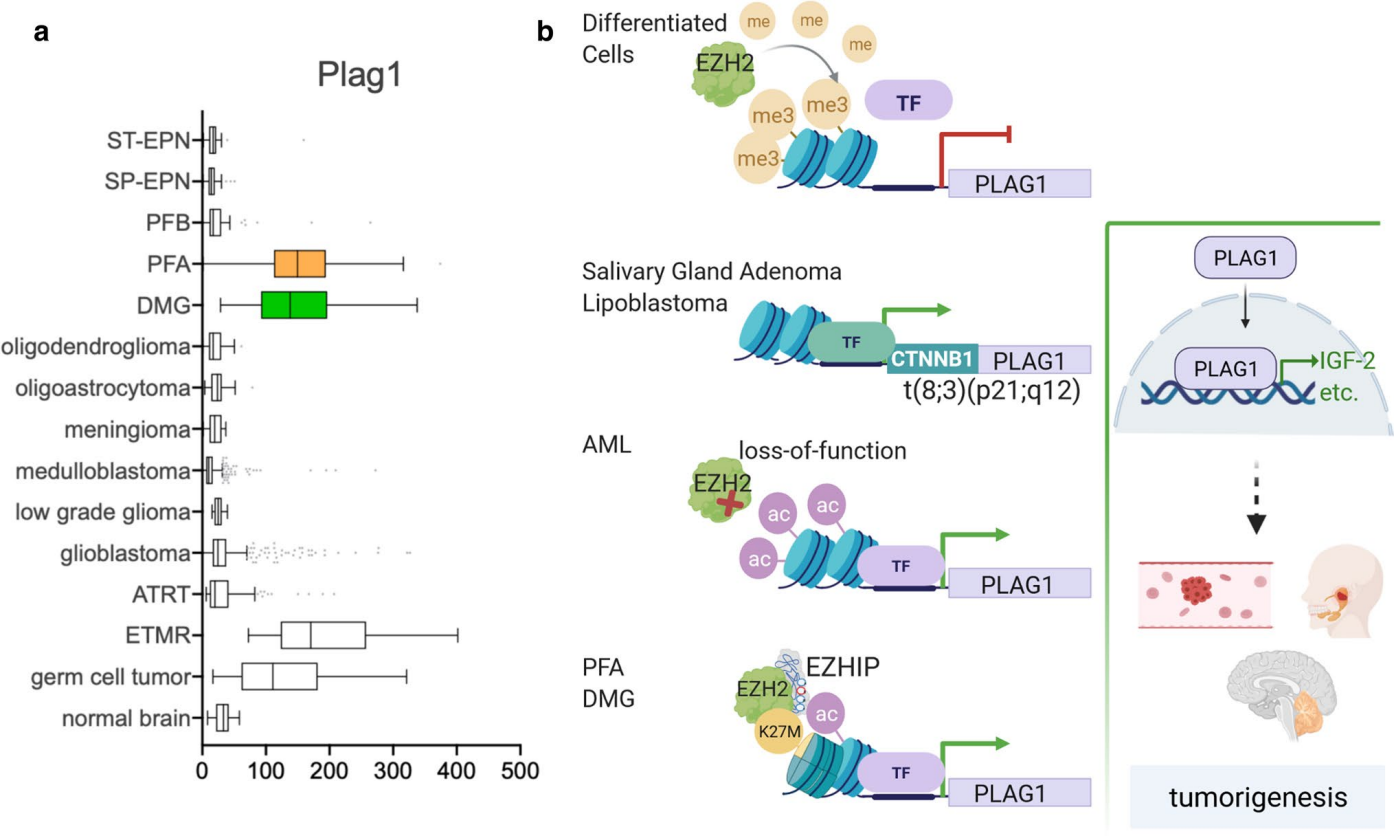
PLAG1 expression in tumorigenesis. **a** *PLAG1* expression levels in different brain tumors, highlighting the high expression in PFA (orange) and DMG (green). **b** Different mechanisms to activate *PLAG1*, which is repressed by EZH2-set H3K27me3 in differentiated cells. Promoter swapping with the *CTNNB1* gene results in fusion genes with expression of *PLAG1* in some solid tumors. In AML, EZH2 mutations can release H3K27me3 at the *PLAG1* locus, as can the presence of EZHIP in PFA and DMG. As a transcription factor, PLAG1 then activates a variety of tumorigenic factors, among them IGF-2

For example, one shared downstream target of PFAs and DMGs with PRC2-impaired tumors is the zinc-finger transcription factor pleomorphic adenoma gene 1 (*PLAG1*), which is de-repressed in EZH2-mutated acute myeloid leukemia (AML) (Fig. 4) [8, 68]. During development, the *PLAG1* promoter gets inactivated epigenetically by H3K27me3 and is not expressed in normal brain or differentiated cells [1, 29]. Upon de-repression, PLAG1 upregulates the expression of growth promoting and cell-fate regulating factors, among them the strong growth enhancer insulin-like growth factor 2 (IGF-2). In benign and malignant solid tumors, such as salivary gland adenomas or lipoblastomas, *PLAG1* is also expressed, but EZH2-independently as the 5’-part of a fusion gene. The t(3;8)(P21;q12) translocation is the most commonly found causing aberration in these diseases, leading to promoter swapping with the constitutively expressed beta-catenin (CTNNB1) [30]. In the brain, PRC2 inhibition by either EZHIP or H3K27M leads to the de-repression of *PLAG1* in PFA, DMG and germ cell tumors. In addition, embryonal tumors with multilayered rosettes (ETMR) express high levels of *PLAG1*, but the regulatory mechanism of *PLAG1* expression in these tumors is still unclear. Other family members of the PLAG1/PLAGL1 transcription factor family caught attention in the context of ependymomas recently. Arabzade et al*.* found the *ZFTA-RELA* fusion to bind to PLAGL1 and PLAGL2-related DNA motifs in ST-EPN tumors. This might be driven by RELA, as its binding sites are commonly flanked by PLAGL1/PLAGL2 recognition sites, indicating a possible co-recruitment [3].

Another noticeable gene of interest is *CDKN2A*, a shared locus with retained H3K27me3 in PFA and DMG coding for the tumor suppressor proteins p16 and p14. P16 is an inhibitor of the cell-cycle regulator CDK4/6 and its silencing allows increased cell-cycle progression. Recently, CDK4/6 was identified as druggable target for ST-EPN, but not for the posterior fossa tumors in a study based on single-cell RNA sequencing and subgroup-specific gene expression [24]. Other studies found a clear reduction of *CDKN2A* transcription levels in EPN with high EZHIP expression [32, 62]. Targeting p16 downstream kinases, H3K27M was found to convey increased sensitivity to CDK4/6 inhibitor treatments in DMG [15].

Besides focusing on specific targets, general epigenetic therapy is an often discussed option for DMG and PFA. Among others, histone deacetylase (HDAC) inhibitors have been tested in several clinical trials for DMG [81]. Ideally, increasing the activating H3K27ac mark releases downregulated tumor suppressing genes. The same goal is driving the idea to use DNA demethylating agents. Even though PFA and DMG are characterized by global DNA hypomethylation and loss of DNA methylation seems to increase high-grade glioma (HGG) tumorigenesis, CpG island hypermethylation can be observed frequently [9, 10, 52]. Treatment of posterior fossa ependymoma xenograft models with DNA demethylating agents was found to decrease tumor burden and increase survival. Also, positive effects of DNA demethylation have been described for *IDH1*-mutant glioma [44, 88].

## Outlook

The identification of EZHIP as a potential tumor driver in PFA spiked a lot of interest and was quickly followed up by intense research into its mechanism of action, sparking hope to finally find answers to all the open questions and challenges in PFA research and patient care. Indeed, a lot was discovered about this new protein. However, the translation into clinically relevant solutions is still one major milestone ahead of the PFA research community.

A shared problem is the lack of relevant tumor models for in vitro and in vivo research. PFA tumors are difficult to take into culture, need high attention and time, if growing at all [46]. Increased numbers of in vitro models are necessary though to validate findings and cover the inter-tumor heterogeneity seen in patient subtypes as well as to substantiate claims from non-PFA cell models. Hand in hand with the need for in vivo models goes the necessity to understand the process of PFA tumor formation. It is still elusive how, where and when EZHIP expression is initiated. Is it the first event happening in the cells on their way to develop into tumor cells? What are prior or additional hits occurring? From DMGs, we can learn that genetic background and timing are essential. Hopefully, future research will attend to this matter. Even though PDX models are being propagated, genetic PFA models would allow for a great increase in opportunities for genetic manipulation, functional understanding and drug testing. With little to no other treatment options besides surgery and radiotherapy available for PFA patients, there is an urgent need to develop other therapy options to improve patient survival. Targeting EZHIP directly might prove difficult, as so far, no own enzymatic activity has been identified. Learning more about the mechanisms of how EZHIP functions, how the mutations affect its function, its interaction partners, and the downstream targets, will hopefully increase the range of potential drug targets for PFA treatment.

## References

[1] Alam S, Zinyk D, Ma L, Schuurmans C (2005). Members of the Plag gene family are expressed in complementary and overlapping regions in the developing murine nervous system. Dev Dyn.

[2] Antin C, Tauziède-Espariat A, Debily MA, Castel D, Grill J, Pagès M (2020). EZHIP is a specific diagnostic biomarker for posterior fossa ependymomas, group PFA and diffuse midline gliomas H3-WT with EZHIP overexpression. Acta Neuropathol Commun.

[3] Arabzade A, Zhao Y, Varadharajan S, Chen H-C, Jessa S, Rivas B (2021). ZFTA-RELA dictates oncogenic transcriptional programs to drive aggressive supratentorial ependymoma. Cancer Discov.

[4] Araki A, Chocholous M, Gojo J, Dorfer C, Czech T, Heinzl H (2016). Chromosome 1q gain and tenascin-C expression are candidate markers to define different risk groups in pediatric posterior fossa ependymoma. Acta Neuropathol Commun.

[5] Ardehali MB, Anselmo A, Cochrane JC, Kundu S, Sadreyev RI, Kingston RE (2017). Polycomb repressive complex 2 methylates elongin a to regulate transcription. Mol Cell.

[6] Bachmann IM, Halvorsen OJ, Collett K, Stefansson IM, Straume O, Haukaas SA (2006). EZH2 expression is associated with high proliferation rate and aggressive tumor subgroups in cutaneous melanoma and cancers of the endometrium, prostate, and breast. J Clin Oncol.

[7] Baroni L, Sundaresan L, Heled A, Coltin H, Pajtler KW, Lin T (2021). Ultra high-risk PFA ependymoma is characterized by loss of chromosome 6q. Neuro Oncol.

[8] Basheer F, Giotopoulos G, Meduri E, Yun H, Mazan M, Sasca D (2019). Contrasting requirements during disease evolution identify EZH2 as a therapeutic target in AML. J Exp Med.

[9] Bayliss J, Mukherjee P, Lu C, Jain SU, Chung C, Martinez D (2016). Lowered H3K27me3 and DNA hypomethylation define poorly prognostic pediatric posterior fossa ependymomas. Sci Transl Med.

[10] Bender S, Tang Y, Lindroth AM, Hovestadt V, Jones DTW, Kool M (2013). Reduced H3K27me3 and DNA hypomethylation are major drivers of gene expression in K27M mutant pediatric high-grade gliomas. Cancer Cell.

[11] Bernstein BE, Mikkelsen TS, Xie X, Kamal M, Huebert DJ, Cuff J (2006). A bivalent chromatin structure marks key developmental genes in embryonic stem cells. Cell.

[12] Bouffet E, Tabori U, Huang A, Bartels U (2009). Ependymoma: lessons from the past, prospects for the future. Child’s Nerv Syst.

[13] Castel D, Kergrohen T, Tauziède-Espariat A, Mackay A, Ghermaoui S, Lechapt E (2020). Histone H3 wild-type DIPG/DMG overexpressing EZHIP extend the spectrum diffuse midline gliomas with PRC2 inhibition beyond H3–K27M mutation. Acta Neuropathol.

[14] Chan KM, Fang D, Gan H, Hashizume R, Yu C, Schroeder M (2013). The histone H3.3K27M mutation in pediatric glioma reprograms H3K27 methylation and gene expression. Genes Dev.

[15] Cordero FJ, Huang Z, Grenier C, He X, Hu G, McLendon RE (2017). Histone H3.3K27M represses p16 to accelerate gliomagenesis in a murine model of DIPG. Mol Cancer Res.

[16] Dewaele B, Przybyl J, Quattrone A, Finalet Ferreiro J, Vanspauwen V, Geerdens E (2014). Identification of a novel, recurrent MBTD1-CXorf67 fusion in low-grade endometrial stromal sarcoma. Int J Cancer.

[17] Djureinovic D, Hallström BM, Horie M, Margareta Mattsson JS, La FL, Fagerberg L (2019). Profiling cancer testis antigens in non-small-cell lung cancer. JCI Insight.

[18] Duan R, Du W, Guo W (2020). EZH2: a novel target for cancer treatment. J Hematol Oncol.

[19] Funato K, Major T, Lewis PW, Allis CD, Tabar V (2014). Use of human embryonic stem cells to model pediatric gliomas with H3.3K27M histone mutation. Science (80-).

[20] Cavalli FMG, Hübner J-M, Sharma T, Luu B, Sill M, Zapotocky M (2018). Heterogeneity within the PF-EPN-B ependymoma subgroup. Acta Neuropathol.

[21] Gan L, Xu M, Hua R, Tan C, Zhang J, Gong Y (2018). The polycomb group protein EZH2 induces epithelial-mesenchymal transition and pluripotent phenotype of gastric cancer cells by binding to PTEN promoter. J Hematol Oncol.

[22] Gatta G, Botta L, Rossi S, Aareleid T, Bielska-Lasota M, Clavel J (2014). Childhood cancer survival in Europe 1999–2007: results of EUROCARE-5—a population-based study. Lancet Oncol.

[23] Ghasemi DR, Sill M, Okonechnikov K, Korshunov A, Yip S, Schutz PW (2019). MYCN amplification drives an aggressive form of spinal ependymoma. Acta Neuropathol.

[24] Gojo J, Englinger B, Jiang L, Hübner JM, Shaw ML, Hack OA (2020). Single-cell RNA-Seq reveals cellular hierarchies and impaired developmental trajectories in pediatric ependymoma. Cancer Cell.

[25] Haag D, Mack N, Goncalves B, da Silva P, Statz B, Clark J (2021). H3.3-K27M drives neural stem cell-specific gliomagenesis in a human iPSC-derived model. Cancer Cell.

[26] Han J, Yu M, Bai Y, Yu J, Jin F, Li C (2020). Elevated CXorf67 expression in PFA ependymomas suppresses DNA repair and sensitizes to PARP inhibitors. Cancer Cell.

[27] Hanna C, Kurian KM, Williams K, Watts C, Jackson A, Carruthers R (2020). Pharmacokinetics, safety, and tolerability of olaparib and temozolomide for recurrent glioblastoma: results of the phase i OPARATIC trial. Neuro Oncol.

[28] Harutyunyan AS, Krug B, Chen H, Papillon-Cavanagh S, Zeinieh M, De Jay N (2019). H3K27M induces defective chromatin spread of PRC2-mediated repressive H3K27me2/me3 and is essential for glioma tumorigenesis. Nat Commun.

[29] Hensen K, Braem C, Declercq J, Van Dyck F, Dewerchin M, Fiette L (2004). Targeted disruption of the murine Plag1 proto-oncogene causes growth retardation and reduced fertility. Dev Growth Differ.

[30] Hess JL, Kossev P (2002). Molecular genetics of benign tumors. Cancer Invest.

[31] Hübner JM, Müller T, Papageorgiou DN, Mauermann M, Krijgsveld J, Russell RB (2019). EZHIP/CXorf67 mimics K27M mutated oncohistones and functions as an intrinsic inhibitor of PRC2 function in aggressive posterior fossa ependymoma. Neuro Oncol.

[32] Jain SU, Do TJ, Lund PJ, Rashoff AQ, Diehl KL, Cieslik M (2019). PFA ependymoma-associated protein EZHIP inhibits PRC2 activity through a H3 K27M-like mechanism. Nat Commun.

[33] Jain SU, Rashoff AQ, Krabbenhoft SD, Hoelper D, Do TJ, Gibson TJ (2020). H3 K27M and EZHIP impede H3K27-methylation spreading by inhibiting allosterically stimulated PRC2. Mol Cell.

[34] Johnson RA, Wright KD, Poppleton H, Mohankumar KM, Finkelstein D, Pounds SB (2010). LETTERS Cross-species genomics matches driver mutations and cell compartments to model ependymoma. Nature.

[35] Justin N, Zhang Y, Tarricone C, Martin SR, Chen S, Underwood E (2016). Structural basis of oncogenic histone H3K27M inhibition of human polycomb repressive complex 2. Nat Commun.

[36] Karremann M, Gielen GH, Hoffmann M, Wiese M, Colditz N, Warmuth-Metz M (2018). Diffuse high-grade gliomas with H3 K27M mutations carry a dismal prognosis independent of tumor location. Neuro Oncol.

[37] Kasper LH, Baker SJ (2020). Invited review: emerging functions of histone H3 mutations in paediatric diffuse high-grade gliomas. Neuropathol Appl Neurobiol.

[38] Khuong-Quang DA, Buczkowicz P, Rakopoulos P, Liu XY, Fontebasso AM, Bouffet E (2012). K27M mutation in histone H3.3 defines clinically and biologically distinct subgroups of pediatric diffuse intrinsic pontine gliomas. Acta Neuropathol.

[39] Kilday J-P, Rahman R, Dyer S, Ridley L, Lowe J, Coyle B (2009). Pediatric ependymoma: biological perspectives. Mol Cancer Res.

[40] Kilday JP, Mitra B, Domerg C, Ward J, Andreiuolo F, Osteso-Ibanez T (2012). Copy number gain of 1q25 predicts poor progression-free survival for pediatric intracranial ependymomas and enables patient risk stratification: a prospective european clinical trial cohort analysis on behalf of the Children’s Cancer Leukaemia Group (CCLG). Clin Cancer Res.

[41] Korshunov A, Witt H, Hielscher T, Benner A, Remke M, Ryzhova M (2010). Molecular staging of intracranial ependymoma in children and adults. J Clin Oncol.

[42] Lewis PW, Müller MM, Koletsky MS, Cordero F, Lin S, Banaszynski LA (2013). Inhibition of PRC2 activity by a gain-of-function H3 mutation found in pediatric glioblastoma. Science (80-).

[43] Louis DN, Perry A, Wesseling P, Brat DJ, Cree IA, Figarella-Branger D (2021). The 2021 WHO classification of tumors of the central nervous system: a summary. Neuro Oncol.

[44] Mack SC, Witt H, Piro RM, Gu L, Zuyderduyn S, Stütz AM (2014). Epigenomic alterations define lethal CIMP-positive ependymomas of infancy. Nature.

[45] Mackay A, Burford A, Carvalho D, Izquierdo E, Fazal-Salom J, Taylor KR (2017). Integrated molecular meta-analysis of 1,000 pediatric high-grade and diffuse intrinsic pontine glioma. Cancer Cell.

[46] Michealraj KA, Kumar SA, Kim LJY, Cavalli FMG, Przelicki D, Wojcik JB (2020). Metabolic regulation of the epigenome drives lethal infantile ependymoma. Cell.

[47] Mikkelsen TS, Ku M, Jaffe DB, Issac B, Lieberman E, Giannoukos G (2007). Genome-wide maps of chromatin state in pluripotent and lineage-committed cells. Nature.

[48] Mohammad F, Weissmann S, Leblanc B, Pandey DP, Højfeldt JW, Comet I (2017). EZH2 is a potential therapeutic target for H3K27M-mutant pediatric gliomas. Nat Med.

[49] Nabirajan A, Sharma A, Rajeshwari M, Boorgula MT, Doddamani R, Garg A (2020). EZH2 inhibitory protein (EZHIP/Cxorf67) expression correlates strongly with H3K27me3 loss in posterior fossa ependymomas and is mutually exclusive with H3K27M mutations. Brain Tumor Pathol.

[50] Nambirajan A, Sharma A, Rajeshwari M, Boorgula MT, Doddamani R, Garg A (2021). EZH2 inhibitory protein (EZHIP/Cxorf67) expression correlates strongly with H3K27me3 loss in posterior fossa ependymomas and is mutually exclusive with H3K27M mutations. Brain Tumor Pathol.

[51] Ning J, Wakimoto H (2020). Therapeutic application of PARP inhibitors in neuro-oncology. Trends Cancer.

[52] Nomura M, Saito K, Aihara K, Nagae G, Yamamoto S, Tatsuno K (2019). DNA demethylation is associated with malignant progression of lower-grade gliomas. Sci Rep.

[53] Ntziachristos P, Tsirigos A, Van Vlierberghe P, Nedjic J, Trimarchi T, Sol Flaherty M (2012). Genetic inactivation of the polycomb repressive complex 2 in T cell acute lymphoblastic leukemia. Nat Med.

[54] Olsen TK, Gorunova L, Meling TR, Micci F, Scheie D, Due-Tønnessen B (2014). Genomic characterization of ependymomas reveals 6q loss as the most common aberration. Oncol Rep.

[55] Ostrom QT, Gittleman H, Liao P, Rouse C, Chen Y, Dowling J (2014). CBTRUS statistical report: primary brain and central nervous system tumors diagnosed in the United States in 2007–2011. Neuro Oncol.

[56] Pajtler KW, Wen J, Sill M, Lin T, Orisme W, Tang B (2018). Molecular heterogeneity and CXorf67 alterations in posterior fossa group A (PFA) ependymomas. Acta Neuropathol.

[57] Pajtler KW, Witt H, Sill M, Jones DTW, Hovestadt V, Kratochwil F (2015). Molecular classification of ependymal tumors across All CNS compartments, histopathological grades, and age groups. Cancer Cell.

[58] Parker M, Mohankumar KM, Punchihewa C, Weinlich R, Dalton JD, Li Y (2014). C11orf95-RELA fusions drive oncogenic NF-κB signalling in ependymoma. Nature.

[59] Paugh BS, Broniscer A, Qu C, Miller CP, Zhang J, Tatevossian RG (2011). Genome-wide analyses identify recurrent amplifications of receptor tyrosine kinases and cell-cycle regulatory genes in diffuse intrinsic pontine glioma. J Clin Oncol.

[60] Paugh BS, Zhu X, Qu C, Endersby R, Diaz AK, Zhang J (2013). Novel oncogenic PDGFRA mutations in pediatric high-grade gliomas. Cancer Res.

[61] Piunti A, Hashizume R, Morgan MA, Bartom ET, Horbinski CM, Marshall SA (2017). Therapeutic targeting of polycomb and BET bromodomain proteins in diffuse intrinsic pontine gliomas. Nat Med.

[62] Piunti A, Smith ER, Morgan MAJ, Ugarenko M, Khaltyan N, Helmin KA (2019). Catacomb: an endogenous inducible gene that antagonizes H3K27 methylation activity of Polycomb repressive complex 2 via an H3K27M-like mechanism. Sci Adv.

[63] Pratt D, Quezado M, Abdullaev Z, Hawes D, Yang F, Garton HJL (2020). Diffuse intrinsic pontine glioma-like tumor with EZHIP expression and molecular features of PFA ependymoma. Acta Neuropathol Commun.

[64] Purdy E, Johnston DL, Bartels U, Fryer C, Carret AS, Crooks B (2014). Ependymoma in children under the age of 3 years: a report from the Canadian Pediatric Brain Tumour Consortium. J Neurooncol.

[65] Ragazzini R, Pérez-Palacios R, Baymaz IH, Diop S, Ancelin K, Zielinski D (2019). EZHIP constrains Polycomb repressive complex 2 activity in germ cells. Nat Commun.

[66] Rajeshwari M, Sharma MC, Kakkar A, Nambirajan A, Suri V, Sarkar C (2016). Evaluation of chromosome 1q gain in intracranial ependymomas. J Neurooncol.

[67] Ryall S, Guzman M, Elbabaa SK, Luu B, Mack SC, Zapotocky M (2017). H3 K27M mutations are extremely rare in posterior fossa group A ependymoma. Child’s Nerv Syst.

[68] Sakai H, Fujii Y, Kuwayama N, Kawaji K, Gotoh Y, Kishi Y (2019). Plag1 regulates neuronal gene expression and neuronal differentiation of neocortical neural progenitor cells. Genes Cells.

[69] Silveira AB, Kasper LH, Fan Y, Jin H, Wu G, Shaw TI (2019). H3.3 K27M depletion increases differentiation and extends latency of diffuse intrinsic pontine glioma growth in vivo. Acta Neuropathol.

[70] Sonnenblick A, De Azambuja E, Azim HA, Piccart M (2015). An update on PARP inhibitors—moving to the adjuvant setting. Nat Rev Clin Oncol.

[71] Sturm D, Witt H, Hovestadt V, Khuong-Quang DA, Jones DTW, Konermann C (2012). Hotspot mutations in H3F3A and IDH1 define distinct epigenetic and biological subgroups of glioblastoma. Cancer Cell.

[72] Sun K, Mikule K, Wang Z, Poon G, Vaidyanathan A, Smith G (2018). A comparative pharmacokinetic study of PARP inhibitors demonstrates favorable properties for niraparib efficacy in preclinical tumor models. Oncotarget.

[73] Swanson AA, Raghunathan A, Jenkins RB, Messing-Jünger M, Pietsch T, Clarke MJ (2019). Spinal cord ependymomas with MYCN amplification show aggressive clinical behavior. J Neuropathol Exp Neurol.

[74] Szulzewsky F, Arora S, Hoellerbauer P, King C, Nathan E, Chan M (2020). Comparison of tumor-associated YAP1 fusions identifies a recurrent set of functions critical for oncogenesis. Genes Dev.

[75] Taylor KR, Mackay A, Truffaux N, Butterfield YS, Morozova O, Philippe C (2014). Recurrent activating ACVR1 mutations in diffuse intrinsic pontine glioma. Nat Genet.

[76] Uhlen M, Fagerberg L, Hallstrom BM, Lindskog C, Oksvold P, Mardinoglu A (2015). Tissue-based map of the human proteome. Science (80-).

[77] Varambally S, Dhanasekaran SM, Zhou M, Barrette TR, Kumar-Sinha C, Sanda MG (2002). The polycomb group protein EZH2 is involved in progression of prostate cancer. Nature.

[78] Venneti S, Garimella MT, Sullivan LM, Martinez D, Huse JT, Heguy A (2013). Evaluation of histone 3 lysine 27 trimethylation (H3K27me3) and enhancer of zest 2 (EZH2) in pediatric glial and glioneuronal tumors shows decreased H3K27me3 in H3F3A K27M mutant glioblastomas. Brain Pathol.

[79] Vladoiu MC, El-Hamamy I, Donovan LK, Farooq H, Holgado BL, Sundaravadanam Y (2019). Childhood cerebellar tumours mirror conserved fetal transcriptional programs. Nature.

[80] Wang Y, Hou N, Cheng X, Zhang J, Tan X, Zhang C (2017). Ezh2 acts as a tumor suppressor in kras-driven lung adenocarcinoma. Int J Biol Sci.

[81] Wierzbicki K, Ravi K, Franson A, Bruzek A, Cantor E, Harris M (2020). Targeting and Therapeutic Monitoring of H3K27M-Mutant Glioma. Curr Oncol Rep.

[82] Witt H, Mack SC, Ryzhova M, Bender S, Sill M, Isserlin R (2011). Delineation of two clinically and molecularly distinct subgroups of posterior fossa ependymoma. Cancer Cell.

[83] Wu G, Broniscer A, McEachron TA, Lu C, Paugh BS, Becksfort J (2012). Somatic histone H3 alterations in pediatric diffuse intrinsic pontine gliomas and non-brainstem glioblastomas. Nat Genet.

[84] Wu G, Diaz AK, Paugh BS, Rankin SL, Ju B, Li Y (2014). The genomic landscape of diffuse intrinsic pontine glioma and pediatric non-brainstem high-grade glioma. Nat Genet.

[85] Wu J, Armstrong TS, Gilbert MR (2016). Biology and management of ependymomas. Neuro Oncol.

[86] Wu W, Klockow JL, Zhang M, Lafortune F, Chang E, Jin L (2021). Glioblastoma multiforme (GBM): an overview of current therapies and mechanisms of resistance. Pharmacol Res.

[87] Xiong Y, Guo Y, Liu Y, Wang H, Gong W, Liu Y (2020). Pamiparib is a potent and selective PARP inhibitor with unique potential for the treatment of brain tumor. Neoplasia (US).

[88] Yamashita AS, Da Costa RM, Borodovsky A, Festuccia WT, Chan T, Riggins GJ (2019). Demethylation and epigenetic modification with 5-azacytidine reduces IDH1 mutant glioma growth in combination with temozolomide. Neuro Oncol.

[89] Zapotocky M, Beera K, Adamski J, Laperierre N, Guger S, Janzen L (2019). Survival and functional outcomes of molecularly defined childhood posterior fossa ependymoma: cure at a cost. Cancer.

[90] Zheng T, Ghasemi DR, Okonechnikov K, Korshunov A, Sill M, Maass KK (2021). Cross-species genomics reveals oncogenic dependencies in ZFTA/C11orf95 fusion-positive supratentorial ependymomas. Cancer Discov.

